# Letter to the editor: exercise stress test as a screening tool for pre-excitation

**DOI:** 10.1093/europace/euaf013

**Published:** 2025-01-17

**Authors:** Marcos Clavero-Adell, Daniel Palanca-Arias, Vicent Modesto I Alapont

**Affiliations:** Pediatric Cardiology Unit, University Hospital Miguel Servet, Paseo de Isabel la Católica 1-3, 50009 Zaragoza, Spain; Faculty of Medicine, Universidad de Zaragoza, Calle Pedro Cerbuna 9, 50009 Zaragoza, Spain; Pediatric Cardiology Unit, University Hospital Miguel Servet, Paseo de Isabel la Católica 1-3, 50009 Zaragoza, Spain; Faculty of Medicine, Universidad de Zaragoza, Calle Pedro Cerbuna 9, 50009 Zaragoza, Spain; Hospital Universitari I Politècnic La Fe, València, Avinguda de Fernando Abril Martorell 106, Quatre Carreres, 46026 Valencia, Spain

We read with great interest the work by Jemtrén *et al*.,^1^ which included subjects with ventricular pre-excitation, both symptomatic and asymptomatic, who underwent an exercise stress test (EST) and an electrophysiological study (EPS). The authors’ aim was to evaluate the sudden loss of pre-excitation during the EST (positive EST) as a diagnostic tool to rule out high-risk atrioventricular accessory pathways (APs), defined as an Accessory Pathway Effective Refractory Period (APERP) or Shortest Pre-Excited RR Interval of <250 ms. They concluded that the sudden loss of pre-excitation during the EST has low sensitivity and a low negative predictive value for ruling out potentially dangerous APs and that EPS should be considered regardless of symptoms.

**Figure 1:**
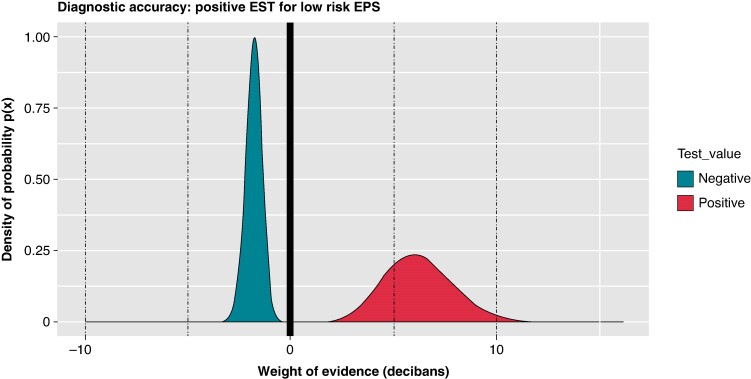
Weight of evidence of positive EST for diagnosis of low risk EPS. WoE + = + 6.16 [95% Credible Interval (Cr Int) = 3.24–9.91]. Pr (WoE+ > 5) = 0.763; Pr (WoE+ > 10) = 0.023. WoE− = −1.78 (95% Cr Int = −2.63 to −1.04). Pr (WoE− < −5) = 0. Computed using MCMC method with a Bayesian model in beta-binomial non-informative model. EPS, electrophysiological study; EST, exercise stress test; WOE, weight of evidence.

This is a prospective, methodologically sound study with a significant number of patients. However, we believe that, from a statistical standpoint, other conclusions can be drawn from its results. According to the data in *Table 1*, the authors report a sensitivity (S) of 40% and specificity (E) of 91% that yields a positive likelihood ratio (LR+) of 4.13 [95% Credible Interval (Cr Int) = 2.11–9.79] and a negative likelihood ratio (LR−) of 0.66 (95% Cr Int = 0.55–0.79). While it is true that the sudden loss of pre-excitation during the EST does not have a strong capacity to identify all high-risk APs, the high E suggests that the finding of pre-excitation loss is highly predictive of the absence of a high-risk AP. Therefore, it could be used as a screening tool, meaning that in subjects with a positive EST, performing an EPS may not be necessary, while in subjects with a negative EST, invasive testing would be required. Additionally, analysing the weight of evidence (WoE) in decibans,^2^ we obtain WoE+ of +6.16 decibans (95% Cr Int = 3.24–9.91) and WoE− of −1.78 decibans (95% Cr Int = −2.63 to −1.04) (*Figure 1*). It further supports the idea that a positive EST has a good ability to rule out the presence of a high-risk AP, but that a negative EST has nevertheless a poor ability to confirm the presence of a high-risk AP.

**Table 1:** Evaluation of the EST as a diagnostic test, with the EPS as the gold standard

	Low-risk EPS (SPERRI/APERP > 250 ms)	High-risk EPS (SPERRI/APERP ≤ 250 ms)	Total
Positive EST (loss of pre-excitation)	39	6	45
Negative EST (loss of pre-excitation)	58	61	119
Total	97	67	164

The values for sensitivity (S), specificity (E), positive likelihood ratio, and negative likelihood ratio are 40%, 91%, 4.13, and 0.66, respectively.

APERP, Accessory Pathway Effective Refractory Period; EPS, electrophysiological study; EST, exercise stress test; SPERRI, Shortest Pre-Excited RR Interval.

In the 2019 European Society of Cardiology clinical guidelines, invasive risk stratification of the AP is recommended (Class I) in individuals with high-risk occupations/hobbies, those participating in competitive athletics, and those patients without ‘low-risk’ characteristics based on non-invasive risk stratification. However, there is controversy, as the guidelines also state that an EPS should be considered to risk-stratify individuals with asymptomatic pre-excitation (Class IIa), while suggesting that non-invasive evaluation of the conducting properties of the AP could be considered in asymptomatic pre-excitation (Class IIb), and that clinical follow-up should be considered for patients with asymptomatic pre-excitation and a low-risk AP at invasive risk stratification (Class IIa).^3^

Therefore, we believe that a new perspective on this study, combined with others with similar results, such as the work by Wackel *et al*.^4^ could provide the EST with value as a screening tool in patients with pre-excitation.
